# The Diversity of Fibrillin Functions: Lessons from the Periodontal Ligament

**DOI:** 10.3390/cells14110764

**Published:** 2025-05-22

**Authors:** Elisabeth Genot, Tala Al Tabosh, Sylvain Catros, Florian Alonso, Damien Le Nihouannen

**Affiliations:** 1 BioTis, U1026, INSERM, University of Bordeaux, F-33000 Bordeaux, France; tala.altabosh@gmail.com (T.A.T.); sylvain.catros@u-bordeaux.fr (S.C.); florian.alonso@inserm.fr (F.A.); damien.le-nihouannen@u-bordeaux.fr (D.L.N.); 2 Department of Oral Surgery, CHU Bordeaux, F-33076 Bordeaux, France

**Keywords:** fibrillin-1, microfibrils, periodontal ligament, Marfan syndrome, connective tissue disease, oxytalan fibers

## Abstract

Marfan syndrome is caused by a mutation in the *FBN1* gene encoding fibrillin-1. This extracellular matrix glycoprotein, which assembles into microfibrils, is best known for its scaffolding role in the production of elastic fibers responsible for connective tissue elasticity and tensile strength. Research into Marfan syndrome mainly focuses on the pathophysiology involved in the degeneration of elastin-rich elastic fibers, which are essential components of the aortic wall. However, fibrillin-1 also exists in elastin-poor (elaunin) or elastin-free (oxytalan) microfibril bundles that were first described in the periodontal ligament (PDL). This dynamic, densely cellular, and highly vascularized tissue anchors teeth in their bone sockets and acts as a protective shock absorber during chewing. Current knowledge suggests that fibrillin microfibrils mechanically support blood vessels in the PDL and ensure their proper functioning. However, many more insights on the roles of fibrillin, especially independently of elastin, can be extracted from this tissue. Here, we review the phenotypic and functional characteristics of the PDL in connection with fibrillin-1, focusing on those related to microvessels. This review aims to shed light on this often-overlooked fibrillin-rich resource as a model for future studies investigating fibrillin functions in health and Marfan disease.

## 1. Introduction

Marfan syndrome (MFS), caused by mutations in the fibrillin-1 gene (15q21), presents with abnormalities of the cardiovascular, ocular, and musculoskeletal systems [1]. Its prevalence is estimated to be 1 in 5000 individuals, which classifies it as a rare disease (OMIM #154700). The constant search for treatments and therapeutic solutions has led to a good understanding of the glycoprotein and its polymerized form into microfibrils. These microfibrils provide a core scaffold on which tropoelastin is deposited to enable the construction of elastic fibers (mature elastic fibers), which appear to be the rationale for fibrillins [2]. However, there are also two other types of elastic fibers: those associated with a lower proportion of elastin, termed elaunin fibers (also referred to as immature elastic fibers), and those that are devoid of elastin, termed oxytalan fibers. Elaunin fibers represent an intermediate and transitional form during the synthesis and maturation of elastic fibers. They are mainly found in tissues that must return to their original shape after being stretched or contracted. Oxytalan fibers are associated with anchoring or connecting structures, where they provide tensile strength and extensibility, but no elasticity [3,4]. Thus, fibrillins also play elastin-independent roles. Information on the different roles of fibrillins in healthy individuals was obtained by examining organ or tissue dysfunctions characteristic of MFS. Mouse models have been important in elucidating these functions within each of the affected tissues and in experimenting with therapeutic solutions.

Since fibrillin-1 is widely distributed in the body, MFS is a multi-systemic genetic disorder. The main tissues affected are the arteries, eyes, lungs, skeleton, and ligaments. The most representative defects in the case of fibrillin-1 mutations appear in the aorta and the eyes. In large arteries, mature elastic fibers—comprising elastin, which represents up to 95% of the dry weight, and fibrillin microfibrils, which account for 5% of the dry weight—are the main constituents of the tunica media. The aneurysms that develop in the aorta of MFS patients show the importance of fibrillin-1 integrity in conferring resistance and resilience to the aortic wall. However, these are not the only properties imparted because recent analyses of a mouse model of MFS showed that arterial defects are not limited to large-caliber arteries or aging arteries, since the small arterioles are already dilated in animals that are a few days old [5]. Hence, the entire arterial network is affected, and arterial enlargement does not solely reflect tissue fatigue.

As for the eye, it is the oxytalan fibers (totally devoid of elastin) of the ciliary zonule (CZ) that are responsible for the strong phenotypic trait [6]. The CZ is a circumferential suspensory ligament that stores the contractile force of the circular ciliary muscle to pull on the lens and thereby modulate its thickness to optimize focus. Made up solely of fibrillin microfibrils, the oxytalan fibers confer resistance, stability, and longevity to the CZ. *FBN1* mutations lead to a weakening of the zonular fibers to the point where they break, causing dislocation of the lens, a condition called ectopia lentis—this represents the predominant ocular complication of MFS.

The three types of elastic fibers are core elements of the skin architecture [7]. The oxytalan fibers penetrate the superficial dermis to connect to elaunin fibers at a specialized basement membrane called the dermal–epidermal junction (DEJ). These, in turn, are anchored onto a thick network of elastin fibers that run in the deep dermis, parallel to the surface of the skin [8]. The sequential arrangement of these three types of elastic fibers, anchored in a basement membrane around lymphatic capillaries, was described in 1990 and named the “fibrillar elastic apparatus” [9]. This network is believed to constitute an elastic device supporting lymph circulation. Likewise, fibrillin microfibrils are thought to play a role in anchoring renal capillaries within the glomerulus basement membrane regions [10], providing mechanical stability to the fine capillary network by preventing excessive stretching under high filtration pressures. The rarefaction of glomerulus capillaries in Marfan patients suggests that the fibrillin-1 defect in the glomerular basement membrane contributes to kidney dysfunction [11]. In these latter situations, fibrillin microfibrils seem to confer extensibility and flexibility to anatomical attachment links.

In addition to their mechanical roles in supporting tissues or connecting functional elements, fibrillins have another major function: giving the extracellular matrix control over the bioavailability of key factors. They serve as a reservoir of regulatory mediators by storing precursors of activatable cytokines, such as Transforming-Growth Factor beta (TGFβ) [12] and certain Bone Morphogenetic Proteins (BMPs) [13]. TGFβ binds to fibrillin-1 indirectly via latent TGFβ-binding proteins (LTBP1, 3, and 4), and this interaction restricts its activation to the relevant regulators. When fibrillin-1 is deficient, this fine-tuned regulation is lost, resulting in TGFβ hyperactivity, which underlies several clinical manifestations of MFS [14]. TGF-β hyperactivity disrupts normal cellular and extracellular matrix signaling by promoting excessive matrix remodeling, inflammation, and altered cell differentiation, which can contribute to the aortic aneurysms and skeletal abnormalities characteristic of MFS [14,15]. In addition to this well-known regulatory action of fibrillins on TGFβ activity, a similar effect seems to operate on BMP10. BMP2, 4, 5, 7, and 10 and Growth Differentiation Factor 5 (GDF5) share a common binding site at the extreme N-terminal region of fibrillin-1 and fibrillin-2 [16]. These interactions involving the BMP prodomain are associated with a conformation of the growth factor complex that is incompatible with binding to its cellular receptor. BMP signaling is activated by the competitive displacement of the prodomain by type II receptors [16,17,18].

A less characterized property of fibrillin is its association with mechanotransduction, the process by which mechanical cues from the cell microenvironment affect intracellular processes. Fibrillins contain the RGD sequence, a ligand motif for many integrins that transmit mechanical signals from the extracellular matrix to the cell, thereby influencing cytoskeletal organization, gene expression, and cell behavior. This interaction not only anchors cells to the extracellular matrix but also enables them to detect and respond to mechanical signals, processes that are essential for tissue homeostasis and development [19,20]. Microfibrils seem to function as extracellular force sensors [21]. When mechanical stresses are applied, fibrillin force-sensitive domains undergo conformational changes. This mechanosensitive mechanism potentially regulates the bioavailability of transforming TGFβ, mentioned above, and ultimately, tissue homeostasis [22]. Thus, changes in topography, compliance, shear, compression, stretching, or movement send signals to the cell, resulting in alterations in gene expression or cell behavior, such as their repositioning or alignment, as shown for fibroblasts [23,24].

More recently, fibrillin-1 was found to be associated with the provisional matrix that instructs invasive endothelial cells, i.e., tip cells, during angiogenesis [25]. In this context, fibrillin-1 is not associated with elastin and wraps endothelial tip cells as a perivascular fibrillin-enriched basement membrane. This role appears distinct from those operating in vascular elastic fibers and may uncover novel fibrillin downstream effectors. At the angiogenic front, fibrillin-1 appears to primarily regulate endothelial cell responses; more specifically, Notch signaling, which is essential for tip cell specification [26]. It should be noted that it is not currently known how the fibrillin associated with the endothelial tip cell’s basement membrane is organized. Indeed, it seems that fibrillin, which is a protein polymerized into microfibrils, could also be found in a non-microfibrillar form [27].

In addition to the functions of fibrillin-1 mentioned above, there is still much to learn about its role in the physiopathology of small vessels. A model that appears well suited to this exploration is that of the periodontal ligament (PDL), which is located between the tooth cementum and the alveolar bone. This tissue can be very informative as it uniquely combines a dense vascularization network and a high fibrillin content with an elastic fiber system containing little or no elastin. Interestingly, oxytalan fibers were first discovered in the PDL [28] and described as protecting its structural integrity, particularly in response to mechanical forces. These fibers are particularly abundant around the vasculature and closely associated with the basement membrane. They are thought to prevent blood vessel collapse and contribute to the regulation of vascular flow. In this review, we explore the literature on PDL as a valuable resource of information on the structural and functional roles of fibrillin, independent of elastin, in association with microvessels, as well as the potential implications for therapeutic innovations for PDL.

## 2. The Periodontal Ligament

### 2.1. Overview

The PDL is the thin layer of specialized connective tissue that attaches the tooth to the alveolar bone. It plays a crucial role in the preservation and function of teeth due to its supporting, nutritional, proprioceptive, and homeostatic properties. It absorbs the mechanical stress of chewing, distributes occlusal forces to prevent bone resorption, and is the main player in orthodontic movement. At the same time, the PDL separates the root of the tooth from the adjacent alveolar bone, preventing them from fusing together, a condition known as dentoalveolar ankylosis. The structure and organization of the PDL ensure tooth stability and adaptability to functional demands. The PDL has been the subject of countless studies, largely because of periodontitis. This inflammation of the PDL, which is caused by specific pathogenic bacteria, is a major health issue worldwide, affecting, to varying degrees, around 30 to 50% of the adult population. Because it is a chronic infectious condition destroying the alveolar bone, the disease can lead to tooth loss and contribute to systemic disorders such as diabetes, cardiovascular disease [29,30], and possibly neurodegenerative diseases [31].

### 2.2. Means of Analysis

Today, basic research on the PDL is mainly based on rodent models, due to the affordability and availability of genetic tools; however, human teeth extracted for orthodontic purposes can also be used. The oxytalan fibers can be visualized by aldehyde–fuchsin staining after oxidation [32,33]. Although this technique does not provide quantitative or structural detail, it is ideal for basic identification and mapping of oxytalan fibers in tissue samples. In addition, fibrillin-1 and fibrillin-2 can be distinguished by immunofluorescent staining [34], which requires high-quality, isoform-specific antibodies, but is essential for studying the distribution, expression, and changes in fibrillin isoforms. The morphology and distribution of the oxytalan fiber meshwork are very similar in humans, mice, and rats [28,35,36]. In the early stages, 2D histological sections and morphometric analysis by transmission electron microscopy were the two main approaches for studying the tissue, with discussions on the biological functions of oxytalan fibers being limited to observations. Nowadays, more advanced techniques allow deeper analysis. These include (i) cryo-electron microscopy, which enables high-resolution visualization of the structures of fibrillin microfibrils in quasi-native states [37] (requires purified samples but is suited to understanding molecular architecture), (ii) atomic force microscopy, which helps characterize the structure of fibrillin-1 microfibrils at the nanoscale level [38] (only examines surface topography but is powerful for mechanical characterization (e.g., elasticity, stiffness) and visualization at the nanoscale), (iii) X-ray diffraction on fibers to evaluate the extensibility and mechanical properties of microfibrils (has lower resolution than Cryo-EM but is useful for understanding mechanical function and structural transitions under strain), and (iv) automated electron tomography to generate three-dimensional reconstructions of isolated microfibrils (computationally intensive but crucial for understanding 3D architecture of microfibril networks). These technical advances have documented the role of calcium in the organization and functionality of microfibrils, as we will see below. In addition, biomechanical tests applied to tissues containing fibrillin microfibrils provide information on their contribution to the tissue’s structural and mechanical properties [39].

### 2.3. Anatomy of the PDL

**Figure 1:**
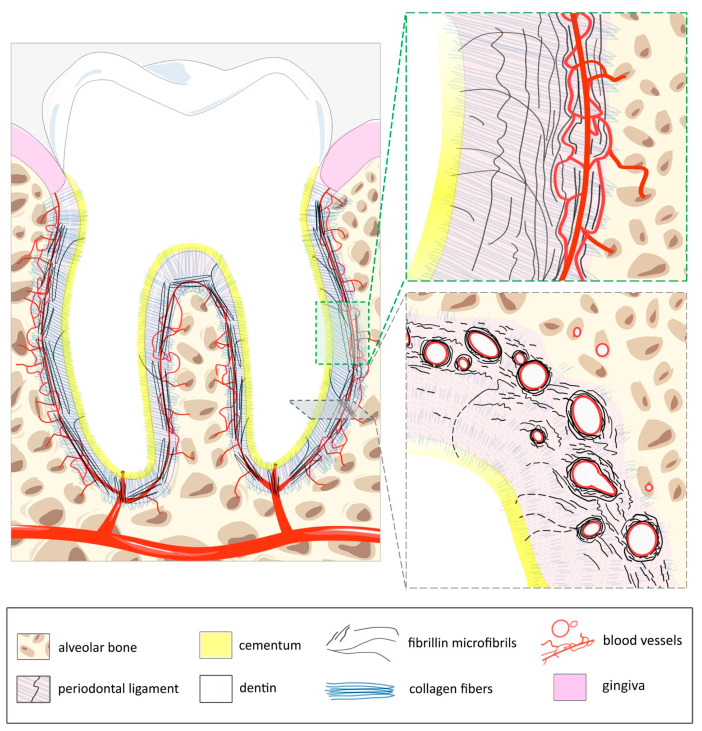
Schematic cross-sectional representation of a tooth, illustrating the organization and spatial relationship between the fibers of the PDL with the more abundant blood vessels on the alveolar bone side (left panel). High magnification views of the two framed regions of the left panel show the sagittal (upper right panel) and transverse (lower right panel) organizations of oxytalan fibers (fibrillin microfibrils) with collagen fibers ending in Sharpey’s fibers (bone perforating fibers). The capillaries and arterioles, embedded in the extracellular matrix, are surrounded by fibrillin fibers. The cementum is a calcified tissue that covers the root and anchors the fibers of the PDL to the tooth. It is produced by cementoblasts, and those that are not trapped in the cementum line up along the cementum surface throughout the entire length of the external covering of the PDL. The dentin is also a calcified tissue, more concentrated in hydroxyapatite crystals than cement. It is covered by enamel on the crown and cementum on the root and surrounds the entire pulp. It provides structural support and transmits sensory stimuli. The gingiva consists of a superficial epithelial layer and a deeper connective tissue layer rich in blood vessels, nerves, and fiber bundles. It plays a crucial role in the innate immune response to infectious inflammation in periodontal tissue. Thus, it is a key mediator in the initiation of periodontal disease.

The PDL develops from the follicular sac that surrounds the embryonic tooth during development. Its width varies with age and functional load, generally ranging from 0.15 to 0.38 mm (Figure 1). It is rich in fibers, with over 80% composed of fibrillar collagen (I and III), which provides most of the structural support. These fibers are arranged in transverse bundles, whose ends, called Sharpey’s fibers, are inserted into both the cementum of the tooth and the alveolar bone [40]. Between groups of collagen fibers, the interstitial space is filled with a network of blood vessels, nerves, and lymphatics. The PDL is highly vascularized for a connective tissue (~20% of the tissue occupied by blood vessels), especially in the apex area [41]. This reflects the high renewal rate of its matrix and cellular components. Blood vessels generally run along the long axis of the tooth with transverse connections and arteriovenous anastomoses [42]. Part of the network is made of fenestrated capillaries (also uncommon for connective tissues), which facilitate gas, fluid, and protein exchange. Venous drainage is achieved by axially directed vessels that flow into a network of large-diameter venules in the apical part of the PDL. Lymphatic vessels typically run parallel to venous drainage. Cell density is high compared to other connective tissues. It contains synthetic cells (fibroblasts, osteoblasts, and cementoblasts), resorptive cells (fibroblasts, osteoclasts, cementoclasts), epithelial rests of Malassez, progenitor cells, and immune cells. Fibroblasts line up along the collagen bundles, wrapping around them with their cytoplasmic processes. Fibroblasts predominate in the PDL, which houses a variety of fibroblast cell populations with different functional characteristics. They produce the structural connective tissue made of the two types of fibers: collagens (mentioned above) and elastic fibers (depicted below), as well as the ground substance, which is an amorphous matrix made of proteoglycans. This highly hydrated matrix contributes to the rigidity of the PDL under compression and to its ability to dissipate the occlusal load. The oxytalan fibers also play a major role in this function. They represent the remaining 15% of the fibers of the PDL and appear to fulfill essential functions for the blood vessels with which they are associated. These functions are presented and discussed below.

### 2.4. Elastic System–Blood Vasculature Coupling in the PDL

When oxytalan fibers were discovered in the PDL, it was also noted that they were closely linked to the vasculature, running parallel to blood vessels [43,44]. This association is evident both during development and in adulthood, and is even more prominent at branching points [45], indicating a supportive role for the vascular network [43,46]. By focusing on oxytalan fibers in the PDL, a lot can be learned about the elastin-independent role of fibrillin in this tissue.

Oxytalan fibers form thick bundles that are regularly arranged and run vertically, mainly parallel to the root surface of the tooth and perpendicular to the horizontal collagen fibers (Figure 1). They are more abundant near the alveolar bone and frequently curve to be inserted into the cementum facing it [47]. On the alveolar bone side, which is more vascularized, the oxytalan fibers surround capillaries individually and attach themselves to their wall, thus enveloping the entire vessel network [35]. Detailed analysis [48] and statistical correlations [49] between blood vessels and oxytalan fibers in a mouse PDL model revealed the existence of two separate populations of oxytalan fibers within the PDL: vascular and non-vascular fibers. For a long time, elastic fibers in the PDL were believed to lack elastin, until Inoue and collaborators reported the presence of elaunin fibers, mainly in the close vicinity of microvessels in the apical region of both the developing and mature PDL of the molars [33,44,50]. The addition of elastin to oxytalan fibers to form these elaunin fibers, is expected to confer elasticity and resilience to regions of the PDL that are more exposed to mechanical stress. Elaunin fibers intertwine with the oxytalan fibers, extending from the cementum to the bone and enveloping the collagen fibers [44]. These interactions could be required for generating a coordinated response to mechanical stress, supporting vascular structures, and maintaining tissue integrity. Oxytalan fibers primarily contribute to tensile strength and anchorage, while elaunin fibers mainly provide elastic cushioning, ensuring that the PDL can withstand and adapt to the mechanical demands of dental functions.

Based on these observations, it is widely accepted that oxytalan fibers strengthen tissue structure by enhancing the cohesion of collagen fibers, thereby resisting distortion. The presence of oxytalan fibers around the periodontal vasculature suggests a protective role during mechanical stress. By preventing the collapse of blood vessels, the fibers likely help in supporting vascular organization and adapting to changes in blood flow impacted by the mechanical forces of mastication [51]. As we will discuss below, these forces, which place considerable strain on the fibers, simultaneously encourage their renewal. The interplay of the oxytalan network with the peripheral nervous system and its juxtaposition with the endothelium of the venules gave rise to the hypothesis that oxytalan fibers might be involved in proprioception. It should be kept in mind that the functional properties attributed to fibrillin in the PDL are largely extrapolated from data obtained from imaging of the PDL and the properties of fibrillin demonstrated in other tissues.

## 3. Fibrillins at Work in the PDL

### 3.1. Fibrillin Isoforms and Functional Domains

**Figure 2:**
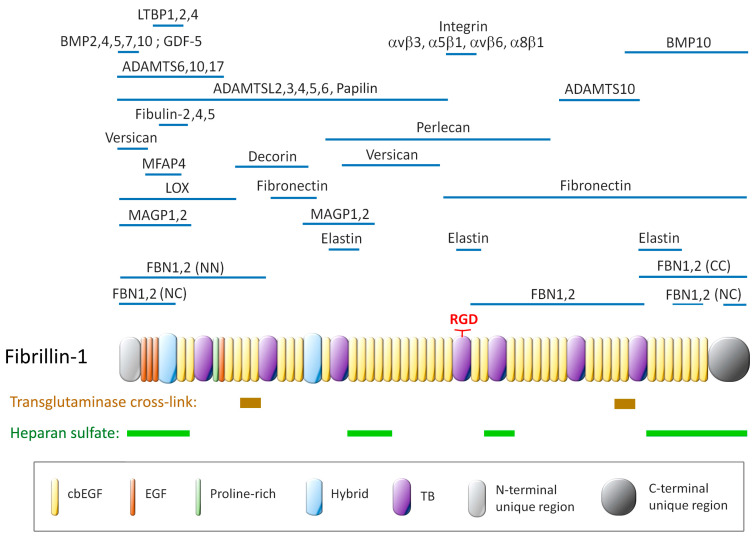
Schematic representation of the modular structure of fibrillin-1 showing the regions of interaction with partner molecules, those that regulate the bioavailability of factors, those involved in the construction of microfibrils or elastin-containing fibers, those with effector functions, and those involved in cell attachment: the RGD sequence which binds integrins and the heparan sulfate binding sites which interact with heparan sulfate proteoglycans. Adapted from [61] and updated from the literature [62,63].

Fibrillin glycoproteins are large, approximately 350 kDa in their processed form, and multimerize to produce microfibrils with a diameter of 12 to 14 nm (Figure 2). There are three isoforms in humans (fibrillin-1, -2, and -3), but only two (fibrillin-1 and -2) in rodents, and these isoforms are encoded by different genes. Fibrillin-3, which is mainly expressed in the fetal stage, has yet to be assessed in this tissue. Fibrillin-1 and fibrillin-2 are both expressed in the PDL, but the two fibrillins are not distributed in a similar manner [34]. Fibrillin-1 localization in the PDL is restricted to the root side of the tooth, where it is detected in thick fibers. On the other hand, fibrillin-2 is more widely distributed in the PDL. At the root side of the tooth, fibrillin-2 is also detected in thick fibers; however, on the alveolar bone side, its signal corresponds to non-fibrous structures. Fibrillin-2 is the first isoform produced during embryogenesis and is found at the core of microfibrils [52]. It is also primarily involved in tissue remodeling [53]. In the PDL, the fibrillin-producing cells are the fibroblasts [54], as these cells are the primary producers of extracellular matrix components. However, isolated endothelial cells also produce fibrillin-1 in vitro [55] and fibrillin of endothelial origin can be detected in vivo in a mouse knock-in model expressing a fluorescent fibrillin-1 reporter construct. Fibrillin-1 derived from endothelial cells is incorporated into elastic fibers in the medial layer of the aortic wall [56]. Thus, it is possible that in the PDL, microfibrils surrounding blood vessels contain fibrillin-1 that is in part derived from the endothelium of the microvasculature.

Fibrillin isoforms have strong homologies, both in their sequence and in their modular organization (Figure 2). Fibrillin-1 harbors 47 epidermal growth factor (EGF)-like domains, 43 of which bind calcium (cbEGF-like domains). These domains are interspersed by TGFβ-binding domains (TB modules) throughout the polypeptide. Two related domains, the hybrid domains (hyb), which have sequences similar to both the TB and cbEGF domains, are found near the N terminus. The highly ordered structure and the high cysteine content (~13%) are distinctive features of fibrillins. Each of the 47 EGF-like domains has three intramolecular disulfide bonds, which enable proper folding and confer stability to the protein by shielding protease-sensitive sites [57]. In addition, each of the 43 cbEGF-type domains binds to a calcium ion through consensus amino acid residues, and this enhances coordination through electrostatic interactions and chelation. Calcium is also involved in protecting fibrillin-1 from digestion by proteases [58]. Whilst the cbEGF domain is a structural motif common to matrix proteins, particularly those involved in withstanding biochemical forces, fibrillin is unique in that it possesses 43 of them [22,59,60]. EGF-like domains are subject to post-translational modifications such as N-linked glycosylation of asparagine residues, O-glycosylation of serine and threonine residues, and hydroxylation of aspartate and asparagine residues. However, post-translational modifications of fibrillin in the PDL are yet to be explored. Another important feature of fibrillin-1 is that it possesses an RGD (Arg-Gly-Asp) sequence capable of binding exposed integrins on the plasma membrane and thus serves as a site for cell attachment. This aspect will be discussed below.

### 3.2. Fibrillin Assembly into Microfibrils

Fibrillins are polymerized into microfibrils at the plasma membrane of the fibrillin-producing cells [61]. Right at the secretion stage, the pro-fibrillins undergo proteolytic processing at both N and C termini by furin convertase; this step is essential for the multimerization process that occurs at the C-terminus. The initial assembly into octamers is facilitated by cell membrane proteoglycans containing heparan sulfate [64]. This multimerization process is also reinforced by the interaction with matrix fibronectin that stabilizes the assembly and guides the interplay with other microfibril proteins. The N- and C-ends of the fibrillin monomers interact with each other, leading to a head-to-tail alignment that is complemented by their parallel and lateral association. Calcium-dependent cbEGF interactions and hydrophobic packing interactions restrict interdomain flexibility, reinforce lateral fibrillin assembly, and thereby shape microfibrils into elongated and rigid rod-like structures. Final maturation of microfibrils occurs by intermolecular transglutaminase-mediated crosslinking. Rotary shadowing studies have revealed that isolated microfibrils appear as beaded filaments with an inter-bead distance of 56 nm. The beaded appearance results from variations in protein density along the fibril, and the 56 nm periodicity reflects the natural spacing of structural repeats within the microfibril. Calcium removal or supplementation causes significant, reversible changes in this periodicity, highlighting the importance of calcium in determining the supramolecular organization of fibrillin microfibrils [65].

The majority of MFS-causing mutations map to cbEGF domains, affecting either cysteine residues that interfere with disulfide bond formation or residues that are critical for calcium binding. Mutations that impair calcium binding result in an incorrect conformation of fibrillin-1, which can no longer fulfill its role because its rigidity and straightness have been compromised [66]. More generally, even with a mutation/deletion, the monomers can still be incorporated into microfibrils, but the resulting overall supramolecular structure is altered [38]. The changes in the geometry of the beads or reduction of their periodicity weaken the overall structure, leading to less resistance to mechanical stress and subsequently to accelerated tissue weakening over time. Likewise, mutations of cysteine residues within EGF-like domains, which lead to the loss of covalent disulfide bridges, result in incorrect protein folding. Consequently, the protein becomes susceptible to proteolysis. In both cases, these mutations result in haploinsufficiency of fibrillin-1.

### 3.3. Fibrillin and Mechanosensing

Mechanotransduction, the process by which mechanical forces are converted into biochemical signals, is another essential function of the PDL, which is routinely exposed to a plethora of such forces. Fibrillin plays a key role in the acquisition of this ability, mediating how cells detect and respond to mechanical stimuli. The forces of stretching, compression, and shearing exerted on the PDL, as well as the associated biochemical modifications like oxidation by reactive oxygen species, damage matrix proteins and make them sensitive to proteolysis [67,68]. In order to adapt and maintain a constant width, the PDL has developed an increased capacity for the production of matrix, which goes hand in hand with a remarkable repair potential, enabling periodontal regeneration. In other words, the same mechanical forces that damage the matrix proteins trigger their remodeling, increasing the number, size, length, and thickness of oxytalan fibers [69]. In line with this, oxytalan fibers are more abundant in the incisors, where orthodontic forces are higher [36]. Interestingly, mechanical loading by applying orthodontic force also increases the number of blood vessels [70,71], once again linking oxytalan fibers to the vasculature. The formation of oxytalan fibers in response to mechanical stimuli can be studied in vitro using a stretching system. Quantification of fibrillin production at the protein and mRNA levels shows that translation efficiency is improved, resulting in increased production of fibrillin bundles in response to stretching [51]. Strain also induces the coalescence of oxytalan fibers in fibroblast cultures [72] by upregulating fibulin-5 [73,74]. Other in vitro experiments using a centrifugation system to exert external pressure and thereby simulate orthodontic forces confirm that a significant time-dependent upregulation of the tropoelastin gene in periodontal fibroblasts occurs under these conditions [75]. Taken together, in vitro studies support the notion that continuous mechanical stimulation of the PDL promotes the replacement of oxytalan fibers.

As mentioned above, the periodicity of fibrillin microfibrils is approximately 56 nm in the relaxed state. The extensibility of microfibrils, up to three times, is demonstrated by the variation in this periodicity of the bead pattern for microfibrils extracted from various tissues [76]. The helicity that resides in each of the cbEGF domains endows them with the ability to compact, and their tandem repetition amplifies the compression at the microfibril level. The basis of the mechano-sensitivity mechanism was revealed by an analysis carried out with the so-called neonatal region of fibrillin-1 (mutations in this region result in the most severe phenotype), composed of eight consecutive cbEGF-like domains. As stated above, each cbEGF-like domain binds to a calcium ion and is stabilized by three disulfide bonds. Under mechanical stress, the calcium binding affinity of the cbEGF domain decreases, turning them into mechanosensitive modules. The disulfide bonds orchestrate the switching activated by calcium. The cbEGF domain stiffness decreases under strain due to mechanosensitive calcium unbinding. Thus, the 3D-folding of these domains endows fibrillin with both extensibility and mechanosensing. Mutations disturbing calcium binding in these domains that are commonly affected in MFS disrupt this mechanosensitive calcium binding, establishing a potential mechanism by which these mutations contribute to disease pathogenesis [22].

### 3.4. Fibrillin Effector Protein-Mediated Functions and Homeostasis

Another functional role of fibrillin-1 microfibrils results from their ability to partner with specific proteins, such as microfibril-associated glycoproteins (MAGPs), latent TGFβ-binding proteins (LTBPs), A Disintegrin And Metalloproteinase with Thrombospondin motifs (ADAMTS) enzymes, and ADAMTS-like (ADAMTSL) proteins [61,77]. As with the proteins involved in the construction of elastic fibers, the binding region of many of these effector proteins has been mapped on fibrillin, often by Surface Plasmon Resonance (SPR) techniques (Figure 2). The binding sites of many of these partners are located within a narrow region towards the N-terminal end of fibrillin, which suggests that not all of them bind to fibrillin at the same time [77]. It can also be expected that the expression of fibrillin effector proteins may be restricted to certain tissues or even be tissue-specific. Furthermore, many of these binding studies were performed with fibrillin fragments, which may not be representative of microfibrils. Emilin-1 [78] and fibulin-5 [73] were found to co-localise with oxytalan fibers assembled by PDL fibroblasts in culture. Fibulin-3, 4, and 5 have been detected in the PDL, although colocalization with fibrillins has not been assessed [79]. The full inventory of proteins associated with fibrillin in the oxytalan and elaunin fibers adjacent to the blood vessels in the PDL is not yet complete.

In addition to their structural properties and their regulatory functions through effector partner proteins, fibrillins directly interact with cells. This interaction occurs through an RGD sequence, which serves as an attachment site for cell-surface integrins. Fibrillin-1 RGD sequence can bind to α5β1, αVβ3, αVβ6, and α8β1 integrins, while fibrillin-2 binds to at least α5β1 and αvβ3 integrins [19,80]. Fibrillins associated with the basement membrane interact with endothelial cells but could also interact with PDL stem cells (PDLSCs). These cells express specific markers such as STRO-1 and CD146, which are characteristic of mesenchymal stem cells [81]. They reside predominantly in close proximity to blood vessels, similar to pericytes [82]. This strategic positioning facilitates their role in maintaining and regenerating periodontal tissues, including the PDL itself, but also alveolar bone and cementum. We already know that fibrillin-1 is a structural component of the bone marrow niche that enables self-renewal and commitment of mesenchymal stem cells, as well as determination of the cellular fate of progenitor cells [83]. In the bone marrow, this generally occurs by modulating TGFβ availability. In the PDL, the situation could be very similar: fibrillin may provide a microenvironment that fulfills the role of a niche, ensuring the maintenance of PDLSC pluripotency. Notch signaling is indispensable for determining cell fate [84], for instance, in maintaining the tip cell phenotype at the angiogenic front [25]. In a scenario where fibrillin-1 contributes to the vascular niche of stem cells around the blood vessels in the PDL, fibrillin-1 deficiency is expected to perturb PDLSC commitment and PDL regeneration of MFS patients. The data obtained with fibrillin cbEGFs show that these domains interact with each other. We cannot exclude the possibility that these domains interact with other proteins that also have numerous cbEGF domains. Notch, like fibrillin, is a mechanosensitive protein, where the mechanical forces between ligand and receptor are necessary to enable the pathway to be activated. These hypotheses on the regeneration of the PDL are worth exploring, as there is much to be gained in terms of developments and prospects for better preservation of this tissue.

## 4. The PDL in Marfan Patients

Due to its high fibrillin-1 content, one could predict that the PDL is weakened by *FBN1* mutations and thus more susceptible to periodontal diseases. Periodontitis induces the loss of PDL and then the alveolar bone due to inflammation related to infection. In patients with MFS, periodontitis is a serious issue that requires the extraction of diseased teeth prior to elective surgical replacement of part of the aorta or mitral valve, to prevent infective endocarditis. Initial studies suggested a higher incidence of periodontitis in MFS patients compared to non-MFS individuals [85]. However, subsequent meta-analyses showed that MFS patients did not exhibit a higher frequency of periodontitis, but rather presented a more severe condition [86]. It should be noted that another factor also comes into play—the fact that the *FBN1* mutation also affects bone growth and morphology. As a result, patients have a narrower palate, resulting in tooth crowding that may also have an impact on periodontitis initiation and development [86,87]. During dental examinations, practitioners recommend specialized care for dental hygiene in MFS patients, which prevents PDL weakness at all levels from an early stage. Another explanation for the fact that microfibril deficiency associated with the fibrillin-1 mutation may not be so significant is that the repair of the oxytalan fibers in the PDL involves fibrillin-2, the fibrillin of regeneration, which is functional in patients with MFS syndrome. Thus, the expected defects in the oxytalan fibers due to the fibrillin-1 deficiency could be compensated by the production of fibrillin-2, as soon as fibrillin-1 deficiency sets in.

To clarify the function of elastic system fibers in the PDL, fibroblasts were isolated from an MFS patient (carrying a mutation in one of the cbEGF domains) with severe periodontitis. These cells were differentiated in vitro, and no major differences were noted among the parameters tested, with the exception of a decrease in osteopontin expression. The cells attached to hydroxyapatite particles were then transplanted subcutaneously into recipient mice. In comparison with PDL cells from a healthy donor, the network of fibrillin microfibrils produced in the implant with PDL cells derived from the MFS patient was not properly organized. Moreover, the cells did not align correctly along these fibers, as in the control [23,24]. These observations show that fibrillin is directly or indirectly required for normal tissue architecture [38]. There is currently very little data available on neovascularization or blood vessel repair in the PDL. However, the process is essential, as it ensures the renewal of the vascular network. In addition, it has recently been shown that fibrillin regulates angiogenesis [25]. It can therefore be expected that angiogenesis will be compromised by fibrillin deficiency, which is inevitably associated with diseased PDL in MFS or with aging. Given the role of fibrillin-1 in angiogenesis [25], it may be instructive to explore these processes in the PDL.

Traditional MFS mouse models have also been used to explore the consequences of *FBN1* mutations on the pathophysiology of PDL. The hyperactivity of TGFß, which is one major consequence of the fibrillin-1 deficit, is also found in the PDL tissue, and it has been hypothesized that TGFß is involved in the onset of periodontal disease [88]. In the MFS mgΔ mouse model, where the *FBN1* gene carries a deletion and its expression is reduced tenfold [89], it was shown that TGFß gives rise to a chronic inflammatory state that results in an increase in IL17 and TNFα. In turn, these cytokines induce the release of metalloproteinases, which are destructive to periodontal tissue. However, TGFß also stimulates extracellular matrix protein production by fibroblasts [54]. Fibrillar collagen production was thus not decreased, but the fibers were thinner and disorganized [23]. Despite increased TGFβ activity, there was also a marked decrease in the expression of periostin, known to regulate collagen fibrillogenesis and maturation [90]. In the *Fbn1*^C1041G/+^ Marfan mouse harboring a missense mutation substituting glycine for cysteine in one cbEGF domain [91], it was shown, using a ligature-induced periodontitis model, that fibrillin-1 insufficiency does not affect the progression of periodontal tissue destruction, but that persistent inflammation delays wound healing and PDL regeneration [92]. The capillaries are dilated in the MFS mgR mouse model (hypomorphic model with 25% of the normal amount of fibrillin-1 in mgR/mgR mice) [93]. This phenotype is reminiscent of that of the lymphatics, as if a kind of “fibrillar elastic apparatus” has been loosened [9]. In addition, close to the basement membrane, there are fewer fibers associated with the blood vessels, accompanied by a high number of apoptotic cells. It is likely that these features are linked and that the phenotype reflects a defect in fibrillin-1-mediated anchorage to the basement membrane [50].

## 5. Therapeutic Advances as a Result of Improved Basic Knowledge of Fibrillin and Fibrillin Microfibrils

Current strategies for treating connective tissue diseases include the application of stem cells or the use of cytokines capable of restoring tissue integrity and cellular activities to reinstate the functionality of diseased tissue. PDL regeneration is also needed for proper tooth implantation after dental trauma. In the field of PDL research, mouse models offer an accessible experimental setup for evaluating innovative approaches for the regeneration of this tissue. The two main models of PDL regeneration are tooth re-implantation and ligature-induced periodontitis, which are widely used due to their reproducibility and straightforward assessment of regenerative outcomes. In the tooth re-implantation model, a tooth is carefully extracted and re-inserted into its socket after a short period ex vivo. This model mimics traumatic dental injury and allows the study of PDL healing, focusing on reattachment of fibers, cementum remodeling, and inflammation resolution. In the ligature-induced periodontitis model, a silk or nylon ligature is tied around the cervical region of a tooth (usually molars), leading to bacterial accumulation and inflammation-induced tissue breakdown. Removal of the ligature initiates the regenerative phase. These models are considered moderately invasive (involving local tissue manipulation but minimal systemic impact) and allow for quantifiable outcomes such as histological scoring, immunostaining of extracellular matrix proteins, and micro-CT analysis of bone and ligament integrity, making them valuable tools for preclinical testing of regenerative therapies.

One of these innovative approaches proposes the administration of matrix components as a complementary therapeutic strategy to existing approaches. The most prominent application aims at alleviating the PDL defects underlying periodontal disease. Although the production of recombinant fibrillin-1 can now be achieved [94], delivering functional fibrillin-1 through gene therapy would be difficult due to the large gene size and complex matrix protein interactions. An alternative option is to use fibrillin extracts, which have conferred a therapeutic benefit when tested in a mouse model of tooth re-implantation [53,54]. Fibrillin prepared from pig aortas was administered in gel form between the bone cavity and the tooth during reimplantation. As a result, root resorption and ankylosis were reduced, while the space between the tooth root and the alveolar bone widened. This space was found to be enriched with periostin and populated with collagen fibers, proving that fibrillin promotes PDL regeneration at least in the microfibril form as prepared and in this model. It should be noted here that the use of fibrillin of animal origin, such as that used in these trials, has well-defined limitations in terms of allergy and compliance with ethical regulations based on the same criteria as other animal products already used in clinical practice. Other strategies for matrix repair using matrix proteins have been carried out with recombinant proteins. Takashi Tsuji’s group is investigating the effect of enriching the diseased PDL with ADAMTSL6β, a microfibril-associated protein that promotes the assembly of fibrillin-1 microfibrils [55,56]. Indeed, ADAMTSL6β rescued microfibril disorder after PDL injury in an MFS mouse model. Interestingly, the overactivation of TGFβ was also partially neutralized after ADAMTSL6β enrichment. These results also suggest a new therapeutic strategy for the treatment of fibrillin deficiency. Last but not least, fibrillin is currently being tested as a candidate for generating a scaffold that can prevent dentoalveolar ankylosis after dental trauma [95].

## 6. Conclusions

In this review, we have explored the literature on PDL as a source of information to explore the potential structural and functional roles of fibrillin in association with microvessels, as well as the observed or expected consequences at the PDL level.

The oxytalan fibers, which make up the CZ, are a material of choice for studying the structural and mechanical properties of fibrillins, as these fibers consist almost exclusively of fibrillin and associated proteins. While oxytalan fibers are enriched in the PDL, the high fibrillin and microfibril content of the PDL has not been fully exploited. This is probably because the fibers in the PDL are embedded in a complex, proteoglycan-rich, highly cellular, and vascularized matrix. By turning this obstacle into an advantage, the study of the PDL—specifically the interaction of fibrillin with cells and the signals delivered to them—should provide a better understanding of other functionalities of fibrillins. Thus, the unique properties of the PDL give this tissue functional attributes that are not reproduced by other tissues. The PDL seems to bring together and combine several different fibrillin functionalities. Given that stem cells are at the forefront of regenerative medicine, the priority is undoubtedly to deepen our understanding of the role of fibrillin as a constituent of the stem cell niche within the PDL.

Tissue engineering technologies have now been developed to achieve the regeneration of periodontal tissue. It seems that therapy by administration of extracellular matrix proteins, supplemented by active recombinant proteins, is critical in the progression of these developments, in particular by neutralizing the events that lead to tissue degradation and/or by promoting the assembly of fibrillin microfibrils [96]. The models of PDL regeneration are moderately invasive and relatively easy to assess. They seem viable for reducing the manifestations of Marfan syndrome in the PDL. The models of PDL regeneration are also particularly suitable for experimenting with therapies intended for the repair of the elastic fiber system in general.

Approaches consisting of enriching the PDL with exogenous fibrillin, or stabilizing or stimulating the assembly of endogenous fibrillin, will enhance the protection of blood vessels that are constantly exposed to mechanical stress. The preservation of the vascular network, as it is a source of nutrients and cell renewal through stem cell differentiation, seems to be vital for limiting PDL degradation. It is an essential condition for the regeneration of these tissues. A better understanding of fibrillin in the PDL should help us increase its regenerative capacities in the event of periodontitis or dental trauma. This research should also benefit efforts aimed at reconstructing an attachment complex around dental bioimplants in order to reduce the risk of infection and restore some of the mechanical properties of the PDL.

## Data Availability

No new data were created or analyzed in this study.

## Abbreviations

The following abbreviations are used in this manuscript:

PDL	Periodontal Ligament
CZ	Ciliary Zonule
TGFβ	Transforming Growth Factor beta
BMP	Bone Morphogenetic Protein
MFS	Marfan Syndrome
